# In vivo confocal microscopy and trachomatous conjunctival scarring: Predictors for clinical progression

**DOI:** 10.1111/ceo.13843

**Published:** 2020-09-16

**Authors:** Jeremy J. Hoffman, Patrick Massae, Helen A. Weiss, William Makupa, Matthew J. Burton, Victor H. Hu

**Affiliations:** 1 International Centre for Eye Health, London School of Hygiene and Tropical Medicine, London, UK; 2 Department of Ophthalmology, Kilimanjaro Christian Medical Centre, Moshi, Tanzania; 3 MRC Tropical Epidemiology Group, London School of Hygiene & Tropical Medicine, London, UK

**Keywords:** confocal microscopy, progression, scarring, trachoma

## Abstract

**Importance:**

In vivo confocal microscopy (IVCM) provides high-resolution images of the ocular surface and has been validated in trachomatous conjunctival scarring.

**Background:**

This study used IVCM to identify parameters associated with clinical scarring progression.

**Design:**

Prospective cohort study.

**Participants:**

A total of 800 participants in Northern Tanzania with trachomatous scarring.

**Methods:**

Participants underwent clinical examination, photography and IVCM at baseline and 24-months. Clinical progression of scarring was defined by comparing baseline and 24-month photographs. Masked grading of IVCM images was used to identify scarring at both time points. Multivariable logistic regression was used to assess factors associated with clinical progression.

**Main Outcome Measures:**

Risk factors associated with clinical scarring progression.

**Results:**

Clinical and IVCM assessment of 800 participants were performed at baseline, with 617 (77.1%) seen at 24-months. Of these, 438 of 617 (71.0%) had gradable IVCM images at both time points and 342 of 438 (78.1%) of these could be graded as showing definite clinical progression or no progression on image comparison. Clinical progression was found to occur in 79 of 342 (23.1%). After adjusting for age and sex, clinical scarring progression was strongly associated with a high IVCM connective tissue organization score at both baseline (odds ratio [OR] = 1.84 for each increase in scarring category; *P* =.002) and 24-months (OR = 1.60; *P* =.02). Dendritiform cells present at 24-months were strongly associated with clinical scarring progression after adjustment (OR = 2.62; *P* =.03).

**Conclusions and Relevance:**

Quantitative IVCM parameters, including connective tissue organization score and the presence of dendritiform cells, are associated with disease progression and may be useful markers in trachoma and other conjunctival fibrotic diseases.

## 1 Introduction

Trachoma is the most common cause of infectious blindness worldwide. It is caused by infection of the conjunctival epithelium with *Chlamydia trachomatis* which initiates an inflammatory reaction producing a mixed papillary and follicular conjunctivitis. This active trachoma is normally found in children who are subsequently at risk of developing conjunctival scarring, entropion, trichiasis, corneal opacity and blindness in later life. There are estimated to be 157 million people globally who live in regions requiring trachoma control interventions, with 1.5 million requiring treatment for trichiasis. In 2017 there were 231 447 who were managed for trachomatous trichiasis.^1^ Around 2.2 million people are visually impaired from trachoma, of whom 1.2 million are blind, the vast majority in low- and middle-income countries.^2,3^ The pathogenesis of trachomatous scarring, however, is poorly understood.^3,4^ Chlamydial infection is rarely found in the cicatricial stages and the factors driving the scarring process need to be elucidated.^5^

In vivo confocal microscopy (IVCM) provides non-invasive, high resolution images of living tissue down to the cellular level. It is based on the optical principle of confocality of the illumination and observation systems, where both are focused on the same focal points.^6^ Any light that is outside of this focal plane is therefore highly suppressed, significantly increasing the resolution. In contrast, the maximum resolution of a light-based bio-microscope system, such as the slit lamp, is considerably limited in comparison due to the amount of scattered light. Due to the high resolution and magnification, the confocal field of view is very small. The confocal microscope therefore rapidly scans the focal point through the tissue and reconstructs an image. This image is parallel to the surface being examined.

IVCM has been used within ophthalmology to study a wide range of ocular surface diseases, specifically in the aid to diagnosing and monitoring microbial keratitis including the detection of fungal hyphae and acanthamoeba cysts, performing endothelial cell counts, and assessing potentially malignant lesions.^6–12^ It is non-invasive, safe and can be repeated.^13,14^ IVCM allows the user to assess the inflammatory cell infiltrate, cellular types and degree of connective tissue scarring. However, interpreting the results can be challenging and there is a lack of standardized, validated grading systems. Interpreting IVCM scans without masking clinical information or other data, as is the case in many studies,^15,16,17^ opens the potential for bias and raises questions over interpretation.

We have previously reported a grading system for the quantitative assessment of IVCM images in scarring trachoma.^18–20^ This grading system had good inter-observer agreement (intra-class coefficient of 0.88), showed that IVCM can be used to quantify connective tissue scarring and measure the degree of inflammatory cell infiltrate.^18–20^ We also found that the presence of trachomatous scarring was strongly associated with the presence of dendritiform cells (DFCs).^19^ This present study looked at the use of this grading system in a cohort of individuals with trachomatous scarring to identify if the IVCM presence of DFC and/or scarring are associated with clinical scarring progression.

## 2 Methods

### 2.1 Ethical permission and subject recruitment

This study was approved jointly by the London School of Hygiene and Tropical Medicine Ethics Committee (Ref. 5128), the Kilimanjaro Christian Medical Centre Ethics Committee (Ref. 203), and the National Ethics Committee of the Tanzanian National Institute of Medical Research (Ref. NIMR/HQ/R.8a/Vol. IX/739). Informed consent was obtained before enrolment of each subject. This work adhered to the tenets of the Declaration of Helsinki.

This study was part of a series of studies on the pathogenesis of trachomatous scarring.^3,18,20–22^ These involved the recruitment of 800 adults with trachomatous conjunctival scarring in trachoma endemic communities in Siha District, Kilimanjaro Region, Northern Tanzania. Children and those with trichiasis were excluded. We attempted IVCM examinations on all consenting individuals. Subjects were then re-examined after 24 months following baseline assessment.

### 2.2 Clinical and photographic assessment

**Figure 1:**
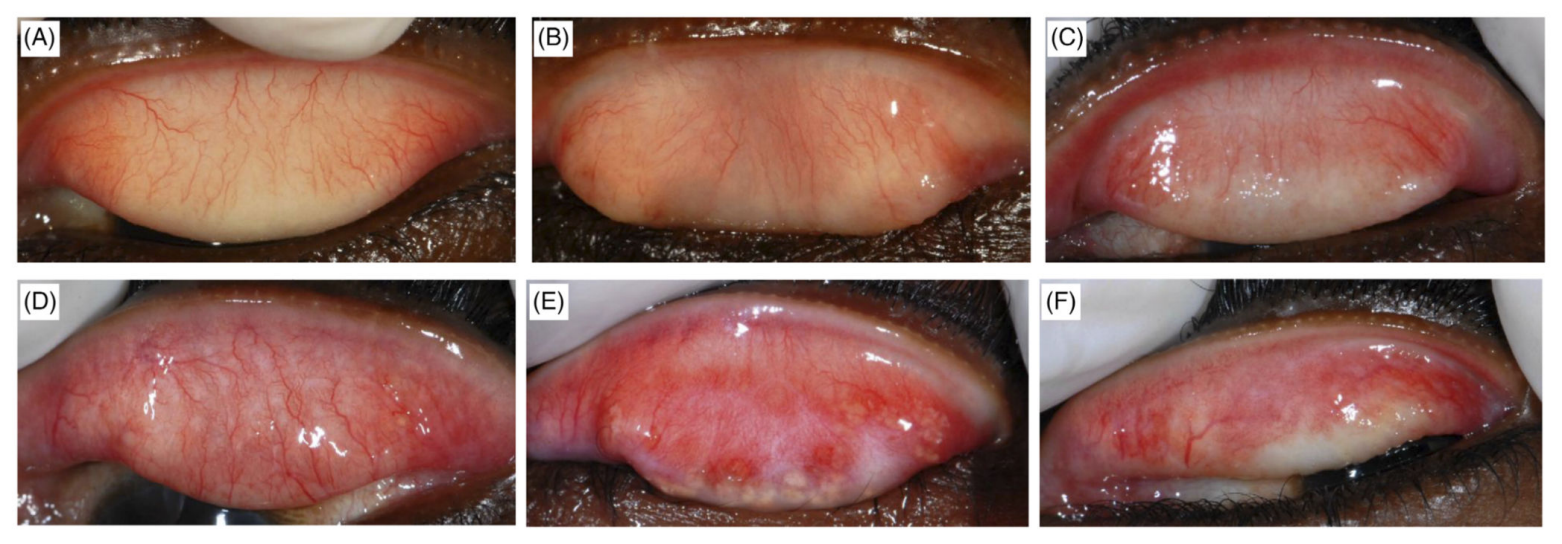
Clinical grading system for trachomatous conjunctival scarring. A, Normal. B, Grade S1a. C, Grade S1b. D, Grade S1c. E, Grade S2. F, Grade S3. *Source:* From Hu et al,^19^ with permission

Subjects were examined at baseline in a dark room or tent with ×2.5 loupes and a bright torch. Signs of trachoma were graded using the 1981 detailed World Health Organization (WHO) grading system, which assesses the upper palpebral conjunctiva for follicles, papillae, and scarring, and grades entropion/trichiasis and corneal opacity.^23^ A modified grading system for assessment of conjunctival scarring was used (Table 1 and Figure 1).^19,22^ A portable slit lamp was used if a more magnified view was needed.

High-resolution digital photographs were taken of the upper tarsal conjunctiva under standardized conditions by the same photographer. Subjects were reexamined and photographed after 24 months. Two ophthalmologists independently identified clinical scarring progression by comparing the high-resolution digital photographs at 24 months to those taken at baseline. To do this, the baseline and 24-month tarsal conjunctival photographs were directly compared side-by-side.

Individuals with progressive scarring, “Progressors,” were defined as those with clear photographic evidence of increased conjunctival scarring at 24-months. “Non-progressors” clearly did not have photographic evidence of scarring progression. Any disparities in progression status were discussed and agreement reached.

### 2.3 IVCM assessment

IVCM examination of the upper tarsal conjunctiva was performed at baseline and again at 24 months using the Heidelberg Retina Tomograph 3 (HRT) in combination with the Rostock Corneal Module (RCM), Heidelberg Engineering GmbH, Dossenheim, Germany using a previously described protocol.^20^ In brief, IVCM was performed to the upper tarsal conjunctiva in the left eye of subjects. Topical anaesthesia was applied to the conjunctival sac (proxymetacaine 0.5%, Chauvin Pharmaceuticals Ltd., Surrey, UK), and the upper eyelid was everted. Scans were taken using the “volume” setting in which 40 coronal images are taken in rapid succession at 2.1-μm intervals from superficial to deep. Scans started at the conjunctival epithelial surface, and the final scan was at a depth of 85 μm. Ten volume scans were taken from different locations from the whole of the tarsal conjunctival surface. It was not possible to record the locations of these scans due to the size of the area being scanned. All IVCM examinations were conducted by the same experienced operator (VH).

The IVCM images were graded for the presence of DFCs and the degree of subepithelial connective tissue organization and scarring, using the previously reported grading system.^19,20^ Although other characteristics can be seen on IVCM such as tissue oedema, papillae and inflammatory infiltrate, these were not graded in this current study. IVCM grading was performed by one of two ophthalmologists (VH or JH) who were masked to the clinical status of the patient. Both graders have significant clinical experience in performing and interpreting corneal and tarsal conjunctival IVCM from large scale clinical studies in Tanzania and Nepal. For every subject, each volume scan was assessed and scored: 0 (normal), 1 (grade 1), 2 (grade 2) or 3 (grade 3) for the connective tissue scarring. The overall IVCM connective tissue organization score for that subject was calculated by dividing the sum of these separate volume scan scores by the number of volume scans graded. Individuals with fewer than three gradable volume scans were excluded from the analysis. To grade the presence or absence of DFCs, all of the available IVCM images were used. Within each volume scan, the section scan with the greatest number of DFCs was used and counted. The mean number of DFCs per volume scan was then calculated. If the mean was equal to, or greater than 1, DFCs were counted as being present.^19,20^

### 2.4 Data analysis

Data were entered into Access 2016 (Microsoft Corp, Redmond, Washington) and analysed using STATA 14.0 (StataCorp LP, College Station, Texas). Multivariable logistic regression models were fitted to assess whether scarring progression was independently associated with the degree of IVCM scarring at baseline or follow-up and the presence or absence of DFCs after adjusting for age. Likelihood ratio tests were used to assess the strength of association of factors with scarring and tests for non-linearity were conducted to assess whether fitting IVCM connective tissue organization score and age increases on a linear scale provided an adequate fit to the data. The *t*-test was used to compare the difference in connective tissue organization score from 24 months and baseline between progressors and non-progressors.

## 3 Results

### 3.1 Study participants

We recruited 800 participants with pre-existing tarsal conjunctival scarring at baseline and re-examined 617 (77.1%) of these at 24 months. Gradable IVCM images, for both baseline and 24-months, were available for 438 of 617 (71.0%) participants. Of these 438, consensus could be reached between the two ophthalmologists performing the photographic grading comparisons, enabling us to confidently determine scarring progression or non-progression in 342 (78.1%), of whom 79 (23.1%) had clinical progression. For validity of the photographic grades, masked grading of a selection of 50 random photographs was performed, giving an intra-class (kappa) coefficient of 0.68. These 342 participants were included in the analysis. There was no difference in baseline characteristics between the 800 participants initially recruited and the 342 included in the analysis. Baseline demographic and clinical scarring data of the 342, by progression status, are shown in Table 2.

We have previously reported good inter-observer agreement for the connective tissue scarring grading (intra-class coefficient of 0.88).^19,20^ Inter-observer agreement was re-assessed for the graders in this current study using a random sample of 50 patients, who were again masked to the clinical appearance. The intra-class correlation co-efficient showed a good agreement of 0.72, that is, 72% of the total variance was due to between individual variation (rather than between observer variation). The percent agreement between the two observers was 95.3%.

### 3.2 IVCM parameters and clinical scarring progression

There was strong evidence of an association between clinical scarring progression at 24-months and an increase in IVCM connective tissue organization score at baseline, after adjusting for age (odds ratio [OR] = 1.81 for each increase in connective tissue organization score; *P* =.002), Table 3. To add face validity, we performed an analysis at 24-months. This, similar to the baseline analysis, demonstrated that an increase in IVCM connective tissue organization score at the 24-month review was associated with clinical scarring progression at 24-months, adjusting for age (adjusted OR = 1.60 for each increase in connective tissue organization score; *P* =.02), Table 3.

There was little evidence that progressive scarring was associated with the detection of DFCs at baseline (Table 3). For face validity purposes, we also looked for an association between DFCs at the 24 month review and clinical scarring progression at 24 months. This confirmed there was evidence that progressive scarring by 24-months was associated with the IVCM detection of DFCs present at the 24-month timepoint (adjusted OR = 2.53; *P* =.03), Table 3.

### 3.3 Baseline and 24-month IVCM connective tissue organization scores

**Figure 2:**
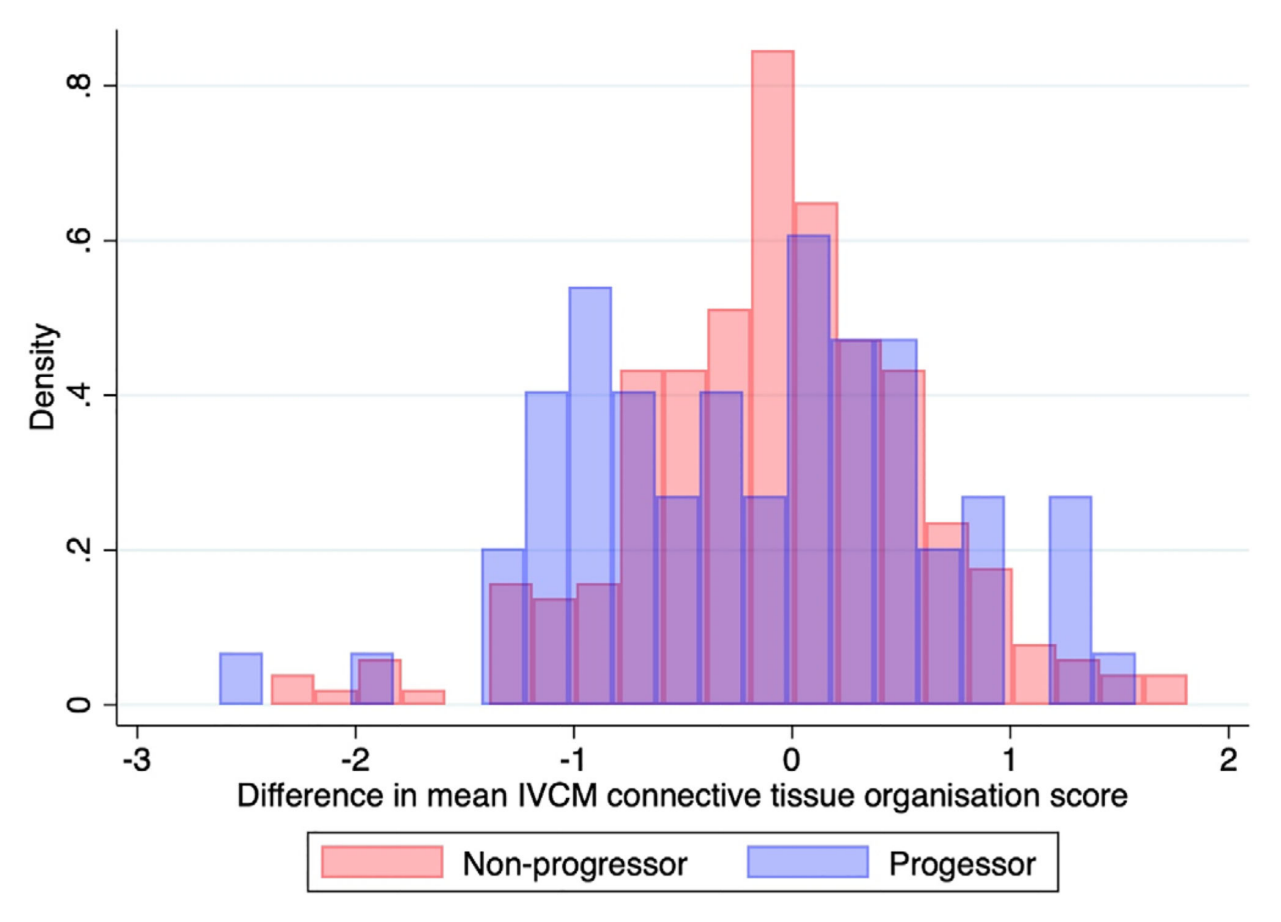
Density histogram showing the difference in the mean in vivo confocal microscopy connective tissue organization score between baseline and 24-month follow-up for non-progressors and progressors

There was no evidence of an association between the baseline and 24-month IVCM connective tissue organization scores. Figure 2 is a histogram showing the difference in the IVCM connective tissue score between 24 months and baseline, showing a normal distribution around 0. The mean difference in this IVCM connective tissue organization score was almost identical for those that showed clinical progression (−0.19) and those that did not (−0.08; *P* =.23).

## 4 Discussion

In this cohort study of individuals with trachomatous scarring examined over 2 years, connective tissue organization score and the presence of DFCs were associated with disease progression.

Increasing IVCM connective tissue organization score at both baseline and at the 24-month follow-up were associated with clinical scarring progression. This suggests that a high IVCM connective tissue organization score at baseline could potentially be used to help identify individuals at increased risk of clinical scarring disease progression. This finding also supports the suggestion that the connective tissue morphology on IVCM may represent subclinical scarring changes in the structure of the tarsal conjunctiva.^19,20^ We have previously reported a strong association between IVCM connective tissue organization score and the histopathological scarring grade.^18^

However, the IVCM connective tissue organization score for individual participants could either increase, remain stable, or decrease, irrespective of whether there had been clinical progression or not. We expected an increase in scarring in IVCM parameters between base-line and 24-months, but Figure 2 demonstrates a normal distribution of difference in scarring between the two time points. There are a number of reasons why this may be the case. Firstly, the area visualized with the confocal microscope is very small (400 by 400 μm) and it is not possible to scan the same area twice nor map the area. We tried to address this by taking 10 random scans over the tarsal conjunctiva at each time point but we speculate that areas of scarred conjunctiva identified at the initial scan may then have been missed at the 24-month scan. It would be interesting to repeat the scans over a longer time period when the progression in clinical scarring is more pronounced. Secondly, although we had good interobserver agreement some variability in the IVCM grading may also have contributed to the apparent lack of IVCM scarring progression. Thirdly, when papillary inflammation was present (as was often the case in scarred participants) the epithelium became thickened and it was difficult to visualize the deeper connective tissue. We kept the HRT-RCM confocal microscope on an automatic gain setting, facilitating subjective interpretation of images rather than quantitative analysis. While this approach worked well overall, dense clinical scarring sometimes gave a rather amorphous appearance on the IVCM image, rather than the typical organized appearance, with little to differentiate the tissue morphology. This may have led to under-grading on our connective tissue organization score and is a potential limitation of assessing the connective tissue with IVCM. However, the results may also reflect the fact that connective tissue analysis with the IVCM according to our protocol may not be showing what we think it is showing and that further research is needed to understand exactly what IVCM is measuring.

We were able to accurately grade 71% of IVCM scans. One of the main reasons for not being able to capture gradable images was lack of participant cooperation. Some participants are unable, or find it difficult, to sit with their chin on the rest and forehead forward. It can also be difficult to evert the upper eyelid and keep it everted and still during the scan. This did not seem to be related to the severity of the scarring as we were certainly able to scan many individuals with advanced clinical grades. Participants were excluded if they had trachomatous trichiasis so tended not to have the very severe scarring which can be seen in advanced trachoma.

We have previously reported that DFCs detected by IVCM in the tarsal conjunctiva are independently associated with trachomatous scarring.^19^ This study showed that DFCs present at the 24 month follow-up were also associated with clinical scarring progression.^19^ Corneal DFCs detected with IVCM are generally thought to be dendritic cells, although this has not been conclusively proven.^24–27^ When comparing IVCM findings with histopathological conjunctival samples from patients with trachoma, there was a discordance between IVCM DFCs and immunohistochemical dendritic cells.^18^ This may have been due to the study methodology, or it is possible that DFCs represent a different cell type. Whatever cell type these structures represent, they appear to be associated with the scarring process in trachoma and it would seem plausible that they are dendritic cells. Dendritic cells are a key antigen presenting cell (APC), providing an important role in immune tolerance and detection and processing of antigens.^28^ Within the eye they are found in corneal and conjunctival tissue. Repeated cycles of immune-mediated inflammation from recurrent infection with *C. trachomatis* are central to trachoma pathogenesis. As a key mediator of immune responses, dendritic cells may play a role in pathogenesis of trachomatous scarring, and may represent an opportunity for disease modification through novel anti-fibrotic agents.^29–33^

There was a similar association for DFCs present at baseline and clinical scarring progression, but this was less convincing after adjusting for other factors. In order for this study to be feasible, this study formed part of a series of studies on the pathogenesis of trachomatous scarring.^19^ As a result, it was not specifically powered to investigate IVCM predictors of clinical scarring progression, which is a limitation. As the number of individuals with DFCs present at either baseline or follow-up were relatively low this study may simply have not had sufficient power to demonstrate a statistically significant effect (a type II error).

IVCM is an expensive tool and not widely available, particularly in lower and middle income countries. Furthermore, it can be technically challenging to perform. However, results from this study suggest that certain confocal parameters (connective tissue organization score and presence of DFCs) provide proof of concept that IVCM could be used as a tool for predicting clinical progression in other cicatricial conjunctival disorders that are more common in developed countires. Similar research into such disorders is therefore required.

We believe this to be the first study to investigate the measurement of IVCM parameters in relation to clinical scarring progression in trachoma. We have demonstrated that increasing IVCM connective tissue organization score at either baseline or follow-up, and the presence of DFCs, are associated with clinical scarring progression after adjusting for age. We did not, however, find IVCM useful for determining whether there had been clinical scarring progression in individual patients. IVCM has potential application to other cicatricial conjunctival disorders, such as Stevens-Johnson syndrome or ocular mucous membrane pemphigoid, and further studies are required to explore its use in these diseases.

## Figures and Tables

**Table 1:** Clinical Scarring Grading System for the tarsal conjunctiva

Grade	Definition
S1	Scarring occupying <1/3 of the upper lid^a^
S1a	One or more pinpoint scars and/or a single line of scarring less than 2 mm in length^b^
S1b	Multiples lines of scarring less than 2 mm in length
S1c	One or more lines/patches of scarring each 2 mm or more in length/maximal dimension
S2	Patches of scarring occupying in surface area ≥ 1/3 but <2/3 of the upper lid
S3	Patches of scarring occupying in surface area ≥ 2/3 of the upper lid

a ”Upper lid” refers to zones 2 and 3 of the everted lid.^23^

b Two millimetre was chosen as this is the approximate width of the lower lid margin, which is readily available for comparison.

**Table 2:** Baseline demographic and clinical scarring data by progression status

Parameter	Clinically did not progress	Clinically progressed	
	n (%)^a^	n (%)^a^	P-value
Sex (N)	263	79	.36
Female	159 (75)	53 (25)	
Male	104 (80)	26 (20)	
Age group (N)	263	79	<.001
18 to 25	38 (88.4)	5 (11.6)	
26 to 35	64 (88.9)	8 (11.1)	
36 to 45	66 (82.5)	14 (17.5)	
46 to 55	39 (72.2)	15 (27.8)	
56 to 65	28 (63.6)	16 (36.4)	
>65	28 (57.1)	21 (42.9)	
Mean age in years (95% CI)	42.1 (40.1-44.1)	52.0 (48.4-55.8)	<.001
Ethnicity (N)	260	79	.24
Maasai	186 (76.9)	56 (23.1)	
Chagga	41 (83.7)	8 (16.3)	
Meru	14 (77.8)	4 (22.2)	
Other	19(63.3)	11 (36.7)	
Baseline conjunctival scarring grade (N)	258	75	<.001
S1a	78 (89.7)	9 (10.3)	
S1b	108 (83.1)	22 (16.9)	
S1c	53 (66.3)	27 (33.8)	
2	9 (47.4)	10 (52.6)	
3	10 (58.8)	7 (41.2)	

Abbreviation: CI, confidence interval.

a Row percentages.

**Table 3:** In vivo confocal microscopy findings at baseline and 24 months follow-up by clinical scarring progression

Parameter	Clinically did not progress n (%)^a^	Clinically progressed n (%)^a^	Unadjusted Association with scarring			Age-adjusted association with scarring		
			OR	95% CI	*P*-value	OR	95% CI	*P*-value
Connective tissue organization score at baseline	N = 257^b^	N = 77^b^	2.15^c^	1.51 to 3.07	<.001	1.81^c^	1.24 to 2.64	.002
<1	118 (84.9)	21 (15.1)						
1 to 2	107 (78.7)	29 (21.3)						
>2 to 3	32 (54.2)	27 (45.8)						
Connective tissue organization score at 24 months	N = 259^b^	N = 76^b^	1.84^c^	1.27 to 2.33	.01	1.57^c^	1.07 to 2.33	.02
<1	137 (82.5)	29 (17.5)						
1 to 2	103 (76.9)	31 (23.1)						
>2 to 3	19 (54.3)	16 (45.7)						
Dendritiform cells at baseline	N = 263^b^	N = 79^b^	1.91	1.00 to 3.66	.05	1.52	0.77 to 3.02	.23
Present	33 (66.0)	17 (34.0)						
Absent	230 (78.8)	62 (21.2)						
Dendritiform cells present at 24 months	N = 263^b^	N = 79^b^	3.04	1.39 to 6.64	.005	2.53	1.11 to 5.77	.03
Present	16 (55.2)	13 (44.8)						
Absent	247 (78.9)	66 (21.1)						

Abbreviation: CI, confidence interval; OR, odds ratio.

a Row percentages;

b The denominator (N) is less for the connective tissue organization score parameter than the dendritiform cells parameter as the protocol required at least three gradable scans for connective tissue grading to be available.

c The OR is the increase with each increase in connective tissue organization score category.
